# Case Report: Complete remission of thymic carcinoma using dose-dense chemotherapy

**DOI:** 10.3389/fonc.2026.1732776

**Published:** 2026-02-26

**Authors:** Mae Shu, Tieying Hou, Stacy M. Rissing, Rohan Maniar, Patrick J. Loehrer

**Affiliations:** 1 Indiana University School of Medicine, Indianapolis, IN, United States; 2 Clinical Pathology & Laboratory Medicine, Indiana University School of Medicine, Indianapolis, IN, United States; 3 Radiology & Imaging Sciences Practice, Indiana University School of Medicine, Indianapolis, IN, United States; 4 Department of Medicine, Indiana University Melvin and Bren Simon Comprehensive Cancer Center, Indianapolis, IN, United States

**Keywords:** breast cancer, case report, dose-dense chemotherapy, neoadjuvant therapy, thymic carcinoma

## Abstract

Thymic epithelial tumors, including thymoma and thymic carcinoma, are rare malignancies of the mediastinum, often associated with poor long-term outcomes in advanced stages. Surgical resection and platinum-based chemotherapy have been the cornerstone of management for resectable and advanced, unresectable disease, respectively, with the use of neoadjuvant chemotherapy alone or chemotherapy combined with radiation in specific cases. We report the case of a patient diagnosed with advanced hormone receptor-positive/HER2-positive breast cancer and a synchronous, advanced thymic carcinoma. After completion of standard-of-care adjuvant dose-dense chemotherapy for her breast cancer, interval imaging revealed marked reduction in the size of the thymic mass with subsequent surgical resection noting a complete pathologic response. The patient remained disease-free for four years before developing recurrence of the thymic carcinoma. This case highlights the potential effectiveness of dose-dense chemotherapy in the treatment of thymic malignancies. Locally advanced and metastatic thymic carcinoma remains challenging to manage, making this patient’s response particularly noteworthy. Given that the therapeutic strategies for thymic tumors have remained largely unchanged in recent years, dose-dense chemotherapy may represent a promising addition to the current treatment paradigm, though further investigation is needed to evaluate its broader applicability.

## Case report

**Figure 1:**
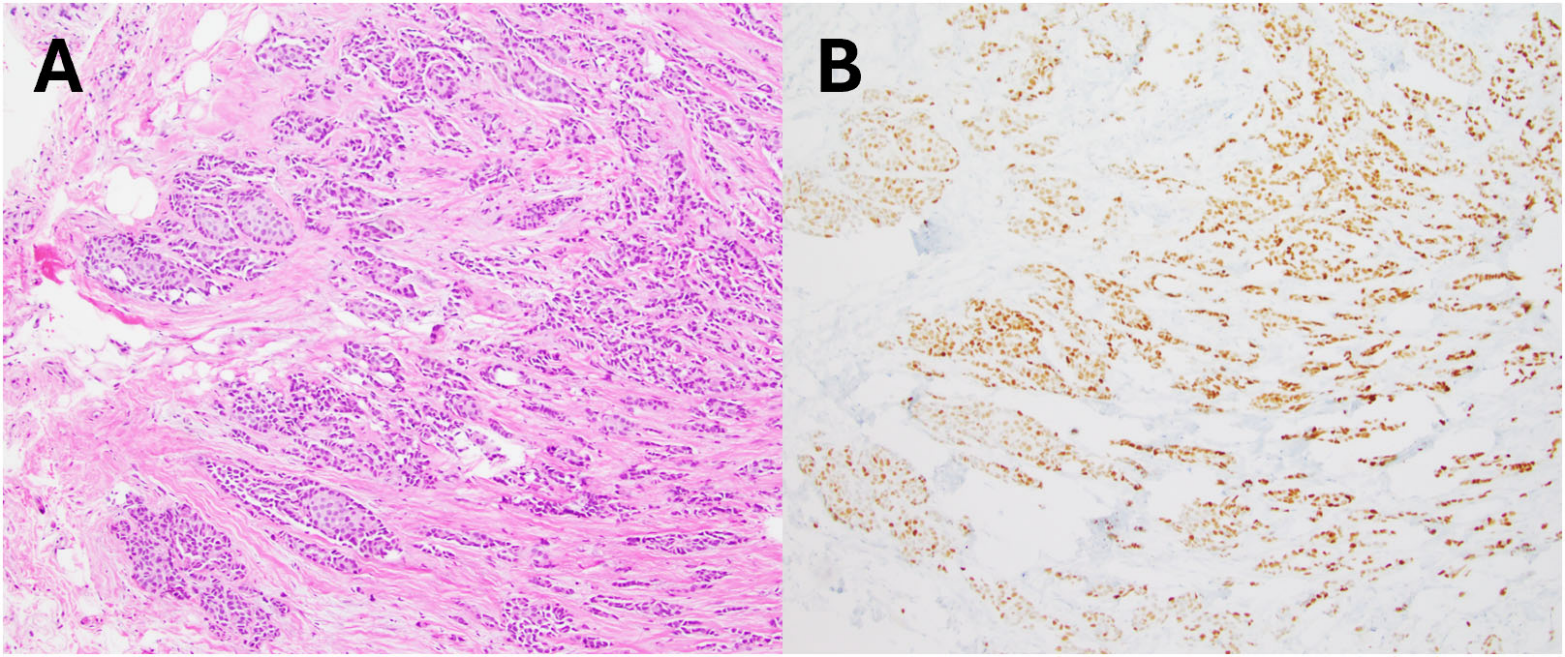
Breast core biopsy. **(A)** H&E section showed an invasive ductal carcinoma, Nottingham grade 2. **(B)** Tumor cells were diffusely positive for progesterone receptor.

A 59-year-old woman with a family history of breast cancer underwent a routine screening mammogram, which revealed an abnormal right breast nodule. Further diagnostic workup with a bilateral breast MRI incidentally revealed a 5.4-cm paramediastinal mass. Genetic testing (Invitae) for BRCA1 and BRCA2 was negative. The patient underwent a partial mastectomy of the right breast tumor with sentinel lymph node dissection positive for metastasis at an outside facility. Histopathological analysis classified the tumor as a moderately differentiated (Nottingham grade 2) invasive ductal carcinoma, staged as pT1cN1aMx (**Figure 1**). The tumor was estrogen receptor (ER)-positive, progesterone receptor (PR)-positive, and HER2-positive (3+ by immunohistochemistry [IHC]). A PET/CT scan post-lumpectomy showed hypermetabolic activity of a soft-tissue mass in the left medial mediastinum (SUV 10.2) for which she presented to our institution for further evaluation.

**Figure 2:**
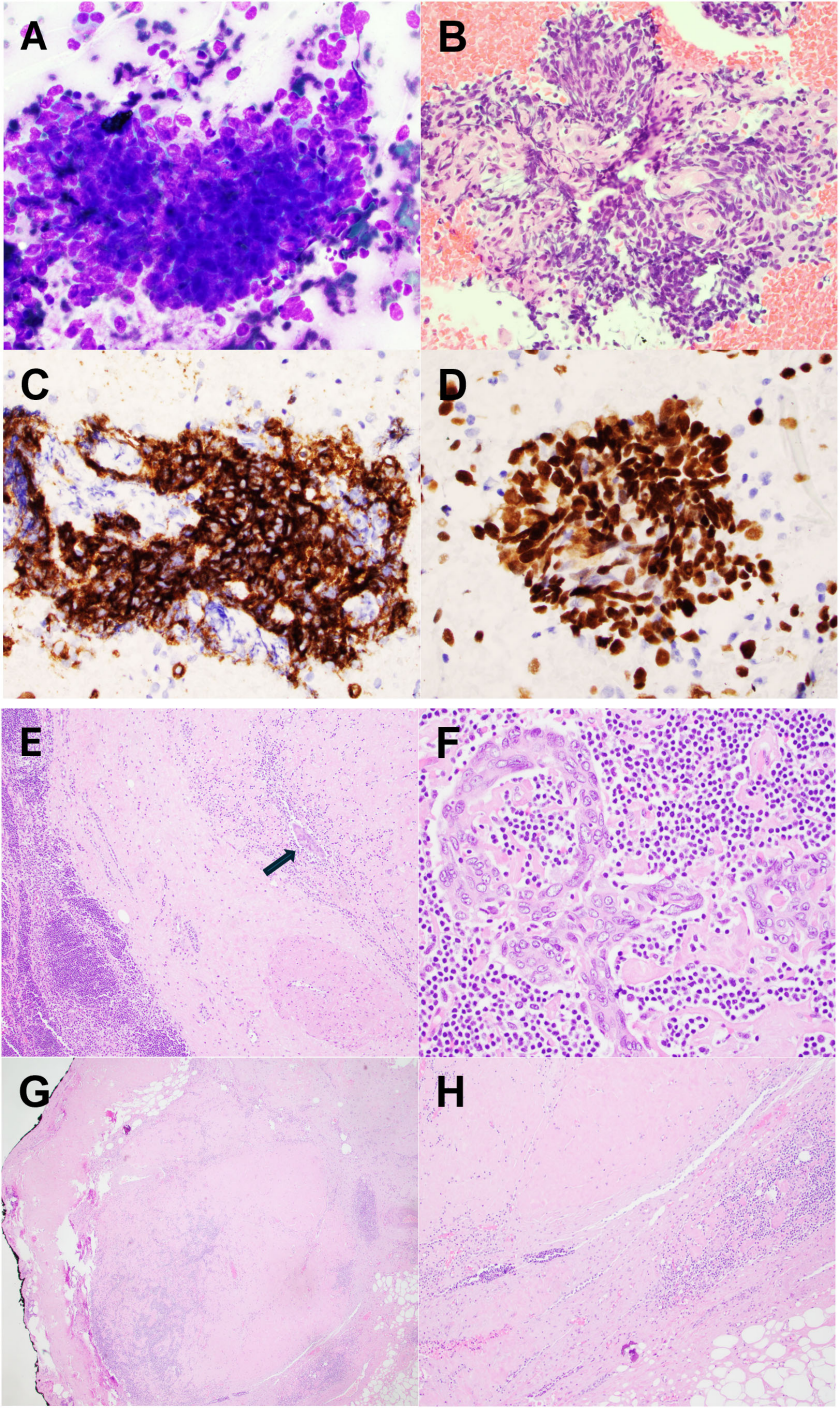
Fine needle aspiration (FNA) from hilar lymph node. **(A)** Direct smear stained by Diff-Quik and H&E section from cell block **(B)** showed basaloid tumor cells with high nuclear-to-cytoplasmic ratio, prominent nucleoli, and severe crush artifact. Tumor cells were diffusely positive for CD117 **(C)** and p40 **(D)**, in keeping with thymic origin. Resection from thymic mass. **(E)** Resection showed a well-defined mass with extensive sclerosis. It contains predominant lymphoid tissue and a scattered epithelial component (arrow). **(F)** Epithelial cells demonstrated cytologic atypia with enlarged nuclei, irregular nuclear membrane, and prominent nucleoli. **(G, H)** Most of the tumor was replaced by sclerosis and calcifications, consistent with treatment-induced changes.

She underwent a biopsy of the hilar lymph node, which revealed a poorly differentiated carcinoma with squamous differentiation. Immunohistochemistry showed the tumor cells were positive for p40 and c-KIT (CD117) (**Figure 2**), but negative for CD5, GLUT-1, and PAX8. CK7 and TTF-1 were also negative. Given the primary tumor’s location and the absence of other lesions in the lungs or head and neck region, thymic carcinoma was considered the leading diagnosis. The specimen was submitted for next-generation sequencing, which demonstrated a molecular signature highly suggestive of thymic origin, with a probability of 96%. The patient was evaluated by thoracic surgery and medical oncology with a recommendation for neoadjuvant therapy given the potential delays in adjuvant therapy for her known breast malignancy and potential overlapping coverage with the planned regimen. The mediastinal mass was closely observed during systemic therapy, with plans for subsequent curative intent surgery of the thymic mass.

The patient received a standard-of-care regimen for breast cancer with dose-dense doxorubicin (60 mg/m^2^) and cyclophosphamide (600 mg/m^2^) [ddAC] for four cycles (8 weeks), followed by paclitaxel (80 mg/m^2^) combined with trastuzumab (8 mg/kg followed by 6 mg/kg) and pertuzumab (840 mg followed by 420 mg) [THP] for 4 cycles (12 weeks) for her breast cancer. Interval imaging after ddAC revealed a significant reduction of the mediastinal mass. The patient then completed THP and subsequent imaging revealed a continued response to therapy with further reduction in the size of the mediastinal mass. She underwent left video-assisted thoracoscopic resection with surgical pathology notable for thymic tissue with sclerosis and no evidence of active malignancy. Epithelial cells showed clustering with high nuclear-to-cytoplasmic ratios suggesting a possibility of an underlying thymoma (WHO type B). Further characterization was limited due to extensive chemotherapy effects. The patient subsequently completed postoperative radiation therapy for her breast cancer followed by anastrozole (which was later switched to exemestane due to intolerance), and pertuzumab and trastuzumab maintenance therapy for 9 months.

**Figure 3:**
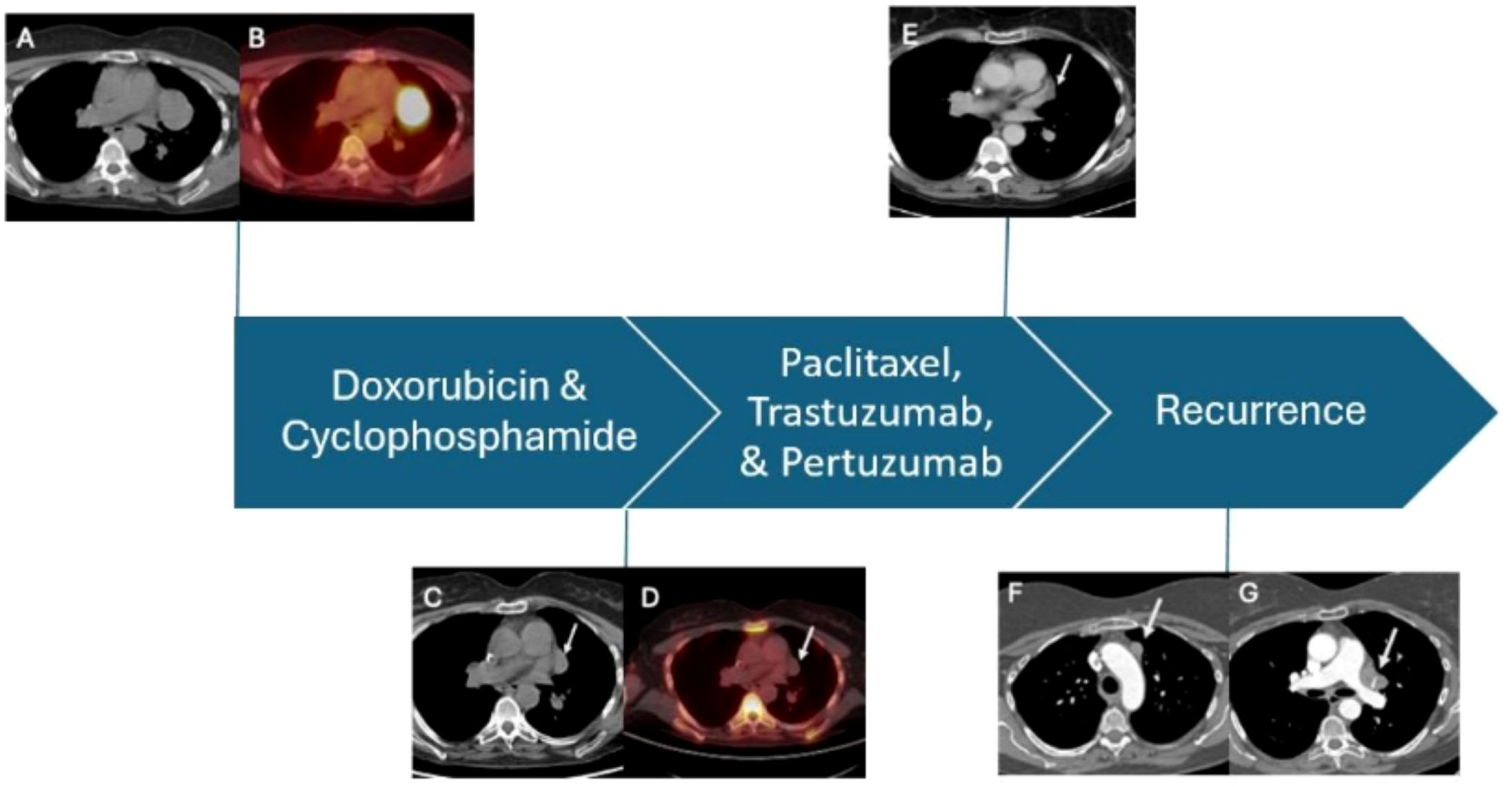
Imaging assessments during the course of treatment. **(A, B)** CT and PET scans prior to adjuvant dose-dense chemotherapy demonstrating a large, hypermetabolic mediastinal mass. **(C, D)** Decrease in the mediastinal mass after completing 4 cycles of doxorubicin and cyclophosphamide as part of dose-dense therapy. **(E)** Lesion decreased in size after completion of paclitaxel, pertuzumab, and trastuzumab prior to resection. **(F, G)** Recurrent mediastinal disease 4 years after definitive surgery.

She remained on active surveillance for her thymic malignancy until interval scans approximately 40 months later revealed a recurrent mediastinal mass with lymphadenopathy. Bronchoscopy with endobronchial ultrasound confirmed left-sided mediastinal lymphadenopathy, and a biopsy revealed metastatic squamous cell carcinoma consistent with her previous thymic carcinoma. The patient was subsequently initiated on single-agent capecitabine with a partial response (**Figure 3**) for about 11 months and stopped due to personal preference.

## Discussion

Thymic epithelial tumors, including thymomas and thymic carcinomas, are rare neoplasms originating from the thymus gland, with an incidence of approximately 0.05 per 100,000 persons per year (1). The standard treatment remains complete surgical resection for patients who are deemed candidates (1). Neoadjuvant therapy is typically administered to reduce tumor burden and inactivate the tumor prior to surgery (1). Response rates to induction chemotherapy range from 30–80%, but durable complete responses are rare (2).

Dose-dense chemotherapy has been extensively studied in breast cancer and has demonstrated clear benefits (3). According to the Goldie-Coldman hypothesis, the early introduction of dose-intensive chemotherapy is more likely to prevent the emergence of resistant tumor clones (4). Studies comparing standard adjuvant chemotherapy to dose-dense regimens in breast cancer have shown improved recurrence-free survival, and distant disease-free survival in patients with breast cancer (3). For example, ddAC followed by paclitaxel administered every two weeks, was found to be superior to the conventional three-week dosing schedule (5). The dose-dense regimen consisted of ddAC (60/600 mg/m^2^) x 4 cycles, followed by paclitaxel (175 mg/m^2^) x 4 cycles every two weeks, with pegfilgrastim (6 mg on day 2) and trastuzumab administered for 1 year (5). There was no significant increase in cardiac toxicity, with no cardiac deaths reported among 70 patients, and only one patient developing congestive heart failure (5).

In thymic cancers, chemotherapy alone is typically not sufficient to completely eradicate the tumor. A group of 22 patients with stage III and IVA thymoma received induction therapy of cyclophosphamide (500 mg/m^2^) on day one, followed by doxorubicin (total 60 mg/m^2^) and cisplatin (total 90 mg/m^2^) on days 1–3 via continuous infusion, and prednisone (100 mg per day) on days 1–5; this cycle was repeated three times every 3–4 weeks and consolidated after surgery with adjuvant chemo-radiotherapy (6). Only 3 (14%) patients had a complete response to induction therapy. While 16 (76%) had a complete resection, only two patients (9%) had complete tumor necrosis or complete pathologic response.

Experience with dose-dense therapy for thymic tumors has been limited. A study conducted in Japan (JCOG 9606) investigated dose-dense chemotherapy in patients with thymoma (7). The regimen was administered over 9 weeks and consisted of cisplatin (25 mg/m^2^) on weeks 1–9; vincristine (1 mg/m^2^) on weeks 1, 2, 4, 6, 8; and doxorubicin (40 mg/m^2^) and etoposide (80 mg/m^2^) on the first three days of weeks 1, 3, 5, 7, and 9 (CODE) and administered with granulocyte colony-stimulating factor support. Thirteen of 21 (62%) eligible patients achieved a partial response while three patients (14%) experienced a complete pathologic response. The study concluded that weekly dose-dense chemotherapy could be safely administered. Notably, nine (39%) patients with initially unresectable thymoma subsequently underwent complete resection after neoadjuvant therapy.

In contrast to the more chemotherapy-sensitive thymoma, thymic carcinoma is generally felt to be less responsive, with complete responses being rare and poorer outcomes comparatively (8). A retrospective analysis of 86 patients with Stage IVA/B thymic carcinoma treated with various regimens, including cisplatin, doxorubicin, vincristine, and cyclophosphamide (ADOC) and cisplatin, doxorubicin and cyclophosphamide (PAC), revealed no complete responses following first-line chemotherapy (8). Moreover, prospective studies of neoadjuvant systemic therapy for advanced thymic carcinomas have been limited with several studies highlighting response rates of 47.6–63% utilizing platinum-based chemotherapy alone or in combination with radiation (9, 10).

In this patient with synchronous HER2-positive breast cancer and thymic carcinoma, ddAC followed by paclitaxel led to a complete pathologic response. Of note, a study evaluated IHC expression on 24 primary squamous cell carcinomas of the thymus showing 58.3% were HER2-positive (11). While the study concluded HER2 amplification is a rare event in thymic carcinoma, protein expression for HER receptors and their ligands are common and may indicate a potential targeted therapy for thymic carcinoma tumors.

## Conclusion

This case highlights the potential efficacy of dose-dense chemotherapy in the treatment of thymic carcinoma. Dose-dense regimens have proven to be effective and safe in both breast cancer and thymoma, but to the best of our knowledge, this may represent the first reported case of a complete pathologic response in thymic carcinoma to dose-dense chemotherapy. While surgical resection remains the cornerstone of treatment for thymic epithelial tumors, neoadjuvant therapy plays a crucial role in improving the likelihood of complete resection and enhancing survival outcomes. Given the aggressive nature of thymic carcinoma, it is rare to achieve a complete response to neoadjuvant therapy and long-term cancer-free progression, highlighting the remarkable outcome in this patient who remained disease-free postoperatively for nearly four years. As the protocols for thymic carcinoma treatment have not significantly changed in recent years, further evaluation of dose-dense chemotherapy is warranted.

## Data Availability

The original contributions presented in the study are included in the article/supplementary material. Further inquiries can be directed to the corresponding author.
